# An SMT-Based Approach for Verifying Binarized Neural Networks

**DOI:** 10.1007/978-3-030-72013-1_11

**Published:** 2021-02-26

**Authors:** Guy Amir, Haoze Wu, Clark Barrett, Guy Katz

**Affiliations:** 8 Eindhoven University of Technology, Eindhoven, The Netherlands; 9 Aalborg University, Aalborg East, Denmark; 10 The Hebrew University of Jerusalem, Jerusalem, Israel; 11 Stanford University, Stanford, USA

## Abstract

Deep learning has emerged as an effective approach for creating modern software systems, with neural networks often surpassing hand-crafted systems. Unfortunately, neural networks are known to suffer from various safety and security issues. Formal verification is a promising avenue for tackling this difficulty, by formally certifying that networks are correct. We propose an SMT-based technique for verifying *binarized neural networks* — a popular kind of neural network, where some weights have been binarized in order to render the neural network more memory and energy efficient, and quicker to evaluate. One novelty of our technique is that it allows the verification of neural networks that include both binarized and non-binarized components. Neural network verification is computationally very difficult, and so we propose here various optimizations, integrated into our SMT procedure as deduction steps, as well as an approach for parallelizing verification queries. We implement our technique as an extension to the Marabou framework, and use it to evaluate the approach on popular binarized neural network architectures.

### 1 Introduction

In recent years, *deep neural networks* (*DNNs*) [21] have revolutionized the state of the art in a variety of tasks, such as image recognition [12,37], text classification [39], and many others. These DNNs, which are artifacts that are generated automatically from a set of training data, generalize very well — i.e., are very successful at handling inputs they had not encountered previously. The success of DNNs is so significant that they are increasingly being incorporated into highly-critical systems, such as autonomous vehicles and aircraft [7,30].

In order to tackle increasingly complex tasks, the size of modern DNNs has also been increasing, sometimes reaching many millions of neurons [46]. Consequently, in some domains, DNN size has become a restricting factor: huge networks have a large memory footprint, and evaluating them consumes both time and energy. Thus, resource-efficient networks are required in order to allow DNNs to be deployed on resource-limited, embedded devices [23,42].

One promising approach for mitigating this problem is via DNN *quantization* [4,27]. Ordinarily, each edge in a DNN has an associated weight, typically stored as a 32-bit floating point number. In a quantized network, these weights are stored using fewer bits. Additionally, the *activation functions* used by the network are also quantized, so that their outputs consist of fewer bits. The network's memory footprint thus becomes significantly smaller, and its evaluation much quicker and cheaper. When the weights and activation function outputs are represented using just a single bit, the resulting network is called a *binarized neural network* (*BNN*) [26]. BNNs are a highly popular variant of a quantized DNN [10,40,56,57], as their computing time can be up to 58 times faster, and their memory footprint 32 times smaller, than that of traditional DNNs [45]. There are also network architectures in which some parts of the network are quantized, and others are not [45]. While quantization leads to some loss of network precision, quantized networks are sufficiently precise in many cases [45].

In recent years, various security and safety issues have been observed in DNNs [33,48]. This has led to the development of a large variety of verification tools and approaches (e.g., [16,25,33,52], and many others). However, most of these approaches have not focused on binarized neural networks, although they are just as vulnerable to safety and security concerns as other DNNs. Recent work has shown that verifying quantized neural networks is PSPACE-hard [24], and that it requires different methods than the ones used for verifying non-quantized DNNs [18]. The few existing approaches that do handle binarized networks focus on the *strictly binarized* case, i.e., on networks where *all* components are binary, and verify them using a SAT solver encoding [29,43]. Neural networks that are only partially binarized [45] cannot be readily encoded as SAT formulas, and thus verifying these networks remains an open problem.

Here, we propose an SMT-based [5] approach and tool for the formal verification of binarized neural networks. We build on top of the Reluplex algorithm [33],[3] and extend it so that it can support the *sign* function,

$$\text{sign}(x) = \begin{cases} x < 0 & -1 \\ x \geq 0 & 1. \end{cases}$$

We show how this extension, when integrated into Reluplex, is sufficient for verifying BNNs. To the best of our knowledge, the approach presented here is the first capable of verifying BNNs that are not strictly binarized. Our technique is implemented as an extension to the open-source Marabou framework [2,34]. We discuss the principles of our approach and the key components of our implementation. We evaluate it both on the XNOR-Net BNN architecture [45], which combines binarized and non-binarized parts, and on a strictly binarized network.

The rest of this paper is organized as follows. In Section 2, we provide the necessary background on DNNs, BNNs, and the SMT-based formal verification of DNNs. Next, we present our SMT-based approach for supporting the sign activation function in Section 3, followed by details on enhancements and optimizations for the approach in Section 4. We discuss the implementation of our tool in Section 5, and its evaluation in Section 6. Related work is discussed in Section 7, and we conclude in Section 8.

[3] [33] is a recent extended version of the original Reluplex paper [31].

### 2 Background

**Deep Neural Networks.** A deep neural network (DNN) is a directed graph, where the nodes (also called neurons) are organized in layers. The first layer is the *input layer*, the last layer is the *output layer*, and the intermediate layers are the *hidden layers*. When the network is evaluated, the input neurons are assigned initial values (e.g., the pixels of an image), and these values are then propagated through the network, layer by layer, all the way to the output layer. The values of the output neurons determine the result returned to the user: often, the neuron with the greatest value corresponds to the output class that is returned. A network is called *feed-forward* if outgoing edges from neurons in layer $i$ can only lead to neurons in layer $j$ if $j > i$. For simplicity, we will assume here that outgoing edges from layer $i$ only lead to the consecutive layer, $i + 1$.

Each layer in the neural network has a *layer type*, which determines how the values of its neurons are computed (using the values of the preceding layer's neurons). One common type is the *weighted sum* layer: neurons in this layer are computed as a linear combination of the values of neurons from the preceding layer, according to predetermined edge weights and biases. Another common type of layer is the *rectified linear unit* (*ReLU*) layer, where each node $y$ is connected to precisely one node $x$ from the preceding layer, and its value is computed by $y = \text{ReLU}(x) = \max(0, x)$. The *max-pooling* layer is also common: each neuron $y$ in this layer is connected to multiple neurons $x_1, \ldots, x_k$ from the preceding layer, and its value is given by $y = \max(x_1, \ldots, x_k)$.

More formally, a DNN $N$ with $k$ inputs and $m$ outputs is a mapping $\mathbb{R}^k \rightarrow \mathbb{R}^m$. It is given as a sequence of layers $L_1, \ldots, L_n$, where $L_1$ and $L_n$ are the input and output layers, respectively. We denote the size of layer $L_i$ as $s_i$, and its individual neurons as $v_i^1, \ldots, v_i^{s_i}$. We use $V_i$ to denote the column vector $[v_i^1, \ldots, v_i^{s_i}]^T$. During evaluation, the input values $V_1$ are given, and $V_2, \ldots, V_n$ are computed iteratively. The network also includes a mapping $T_N : \mathbb{N} \rightarrow \mathcal{T}$, such that $T(i)$ indicates the *type* of hidden layer $i$. For our purposes, we focus on layer types $\mathcal{T} = \{\text{weighted sum}, \text{ReLU}, \text{max}\}$, but of course other types could be included. If $T_n(i) = \text{weighted sum}$, then layer $L_i$ has a weight matrix $W_i$ of dimensions $s_i \times s_{i-1}$ and a bias vector $B_i$ of size $s_i$, and its values are computed as $V_i = W_i \cdot V_{i-1} + B_i$. For $T_n(i) = \text{ReLU}$, the ReLU function is applied to each neuron, i.e. $v_i^j = \text{ReLU}(v_{i-1}^j)$ (we required that $s_i = s_{i-1}$ in this case). If $T_n(i) = \max$, then each neuron $v_i^j$ in layer $L_i$ has a list *src* of source indices, and its value is computed as $v_i^j = \max_{k \in src} v_{i-1}^k$.

A simple illustration appears in Fig. 1. This network has a weighted sum layer and a ReLU layer as its hidden layers, and a weighted sum layer as its output layer. For the weighted sum layers, the weights and biases are listed in the figure. On input $V_1 = [1, 2]^T$, the first

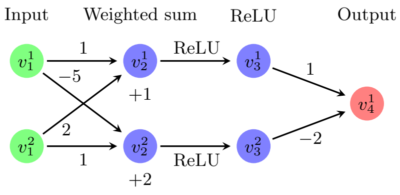

Fig. 1: A toy DNN.

layer's neurons evaluate to $V_2 = [6, -1]^T$. After ReLUs are applied, we get $V_3 = [6, 0]^T$, and finally the output is $V_4 = [6]$.

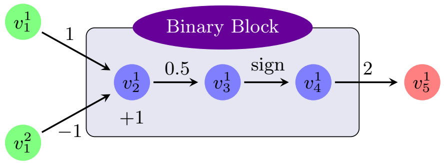

Fig. 2: A toy BNN with a single binary block composed of three layers: a weighted sum layer, a batch normalization layer, and a sign layer.

**Binarized Neural Networks.** In a *binarized neural network* (*BNN*), the layers are typically organized into binary *blocks*, regarded as units with binary inputs and outputs. Following the definitions of Hubara et al. [26] and Narodytska et al. [43], a binary block is comprised of three layers: (i) a *weighted sum* layer, where each entry of the weight matrix $W$ is either 1 or $-1$; (ii) a *batch normalization* layer, which normalizes the values from its preceding layer (this layer can be regarded as a weighted sum layer, where the weight matrix $W$ has real-valued entries in its diagonal, and 0 for all other entries); and (iii) a *sign* layer, which applies the sign function to each neuron in the preceding layer. Because each block ends with a sign layer, its output is always a binary vector, i.e. a vector whose entries are $\pm 1$. Thus, when several binary blocks are concatenated, the inputs and outputs of each block are always binary. Here, we call a network *strictly binarized* if it is composed solely of binary blocks (except for the output layer). If the network contains binary blocks but also additional layers (e.g., ReLU layers), we say that it is a *partially binarized* neural network. BNNs can be made to fit into our definitions by extending the set $\mathcal{T}$ to include the sign function. An example appears in Fig. 2; for input $V_1 = [-1, 3]^T$, the network's output is $V_5 = [-2]$.

**SMT-Based Verification of Deep Neural Networks.** Given a DNN $N$ that transforms an input vector $x$ into an output vector $y = N(x)$, a pre-condition $P$ on $x$, and a post-condition $Q$ on $y$, the *DNN verification problem* [33] is to determine whether there exists a concrete input $x_0$ such that $P(x_0) \wedge Q(N(x_0))$. Typically, $Q$ represents an undesirable output of the DNN, and so the existence of such an $x_0$ constitutes a counterexample. A sound and complete verification engine should return a suitable $x_0$ if the problem is satisfiable (`SAT`), or reply that it is unsatisfiable (`UNSAT`). As in most DNN verification literature, we will restrict ourselves to the case where $P$ and $Q$ are conjunctions of linear constraints over the input and output neurons, respectively [16, 33, 52].

Here, we focus on an SMT-based approach for DNN verification, which was introduced in the Reluplex algorithm [33] and extended in the Marabou framework [2, 34]. It entails regarding the DNN's node values as variables, and the verification query as a set of constraints on these variables. The solver's goal is to find an assignment of the DNN's nodes that satisfies $P$ and $Q$. The constraints are partitioned into two sets: *linear constraints*, i.e. equations and variable lower and upper bounds, which include the input constraints in $P$, the output constraints in $Q$, and the weighted sum layers within the network; and

*piecewise-linear constraints*, which include the activation function constraints, such as ReLU or max constraints. The linear constraints are easier to solve (specifically, they can be phrased as a linear program [6], solvable in polynomial time); whereas the piecewise-linear constraints are more difficult, and render the problem NP-complete [33]. We observe that sign constraints are also piecewise-linear.

In Reluplex, the linear constraints are solved iteratively, using a variant of the Simplex algorithm [13]. Specifically, Reluplex maintains a variable assignment, and iteratively corrects the assignments of variables that violate a linear constraint. Once the linear constraints are satisfied, Reluplex attempts to correct any violated piecewise-linear constraints — again by making iterative adjustments to the assignment. If these steps re-introduce violations in the linear constraints, these constraints are addressed again. Often, this process converges; but if it does not, Reluplex performs a *case split*, which transforms one piecewise-linear constraint into a disjunction of linear constraints. Then, one of the disjuncts is applied and the others are stored, and the solving process continues; and if UNSAT is reached, Reluplex backtracks, removes the disjunct it has applied and applies a different disjunct instead. The process terminates either when one of the search paths returns SAT (the entire query is SAT), or when they all return UNSAT (the entire query is UNSAT). It is desirable to perform as few case splits as possible, as they significantly enlarge the search space to be explored.

The Reluplex algorithm is formally defined as a sound and complete calculus of derivation rules [33]. We omit here the derivation rules aimed at solving the linear constraints, and bring only the rules aimed at addressing the piecewise-linear constraints; specifically, ReLU constraints [33]. These derivation rules are given in Fig. 3, where: (i) $\mathcal{X}$ is the set of all variables in the query; (ii) $R$ is the set of all ReLU pairs; i.e., $\langle b, f\rangle \in R$ implies that it should hold that $f = \text{ReLU}(b)$; (iii) $\alpha$ is the current assignment, mapping variables to real values; (iv) $l$ and $u$ map variables to their current lower and upper bounds, respectively; and (v) the $\textit{update}(\alpha, x, v)$ procedure changes the current assignment $\alpha$ by setting the value of $x$ to $v$. The $\mathsf{ReluCorrect}_b$ and $\mathsf{ReluCorrect}_f$ rules are used for correcting an assignment in which a ReLU constraint is currently violated, by adjusting either the value of $b$ or $f$, respectively. The ReluSplit rule transforms a ReLU constraint into a disjunction, by forcing either $b$'s lower bound to be non-negative, or its upper bound to be non-positive. This forces the constraint into either its active phase (the identity function) or its inactive phase (the zero function). In the case when we guess that a ReLU is active, we also apply the *addEq* operation to add the equation $f = b$, in order to make sure the ReLU is satisfied in the active phase. The Success rule terminates the search procedure when all variable assignments are within their bounds (i.e., all linear constraints hold), and all ReLU constraints are satisfied. The rule for reaching an UNSAT conclusion is part of the linear constraint derivation rules which are not depicted; see [33] for additional details.

The aforementioned derivation rules describe a *search* procedure: the solver incrementally constructs a satisfying assignment, and performs case splitting

$$\text{ReluCorrect}_b \ \frac{\langle b, f\rangle \in R, \quad \alpha(f) \neq \text{ReLU}(\alpha(b))}{\alpha := update(\alpha, b, \alpha(f))} \qquad \text{ReluCorrect}_f \ \frac{\langle b, f\rangle \in R, \quad \alpha(f) \neq \text{ReLU}(\alpha(b))}{\alpha := update(\alpha, f, \text{ReLU}(\alpha(b)))}$$

$$\text{ReluSplit} \ \frac{\langle b, f\rangle \in R}{\begin{array}{l} u(b) := \min(u(b), 0), \\ l(f) := \max(l(f), 0), \\ u(f) := \min(u(f), 0) \end{array} \qquad \begin{array}{c} l(b) := \max(l(b), 0), \\ addEq(f = b) \end{array}}$$

$$\text{Success} \ \frac{\forall x \in \mathcal{X}.\ l(x) \leq \alpha(x) \leq u(x), \quad \forall \langle b, f\rangle \in R.\ \alpha(f) = \text{ReLU}(\alpha(b))}{\texttt{SAT}}$$

Fig. 3: Derivation rules for the Reluplex algorithm (simplified; see [33] for more details).

when needed. Another key ingredient in modern SMT solvers is *deduction* steps, aimed at narrowing down the search space by ruling out possible case splits. In this context, deductions are aimed at obtaining tighter bounds for variables: i.e., finding greater values for $l(x)$ and smaller values for $u(x)$ for each variable $x \in \mathcal{X}$. These bounds can indeed remove case splits by fixing activation functions into one of their phases; for example, if $f = \text{ReLU}(b)$ and we deduce that $b \geq 3$, we know that the ReLU is in its active phase, and no case split is required. We provide additional details on some of these deduction steps in Section 4.

### 3 Extending Reluplex to Support Sign Constraints

In order to extend Reluplex to support sign constraints, we follow a similar approach to how ReLUs are handled. We encode every sign constraint $f = \text{sign}(b)$ as two separate variables, $f$ and $b$. Variable $b$ represents the input to the sign function, whereas $f$ represents the sign's output. In the toy example from Fig. 2, $b$ will represent the assignment for neuron $v_3^1$, and $f$ will represent $v_4^1$.

Initially, a sign constraint poses no bound constraints over $b$, i.e. $l(b) = -\infty$ and $u(b) = \infty$. Because the values of $f$ are always $\pm 1$, we set $l(f) = -1$ and $u(f) = 1$. If, during the search and deduction process, tighter bounds are discovered that imply that $b \geq 0$ or $f > -1$, we say that the sign constraint has been fixed to the *positive* phase; in this case, it can be regarded as a linear constraint, namely $b \geq 0 \wedge f = 1$. Likewise, if it is discovered that $b < 0$ or $f < 1$, the constraint is fixed to the *negative* phase, and is regarded as $b < 0 \wedge f = -1$. If neither case applies, we say that the constraint's phase has not yet been fixed.

In each iteration of the search procedure, a violated constraint is selected and corrected, by altering the variable assignment. A violated sign constraint is corrected by assigning $f$ the appropriate value: $-1$ if the current assignment of $b$ is negative, and 1 otherwise. Case splits (which are needed to ensure completeness and termination) are handled similarly to the ReLU case: we allow the solver to assert that a sign constraint is in either the positive or negative phase, and then backtrack and flip that assertion if the search hits a dead-end.

More formally, we define this extension to Reluplex by modifying the derivation rules described in Fig. 3 as follows. The rules for handling linear con-

$$\mathsf{SignCorrect}_{-}\quad \frac{\langle b, f\rangle \in S,\quad \alpha(b) < 0,\quad \alpha(f) \neq -1}{\alpha := \mathit{update}(\alpha, f, -1)} \qquad \mathsf{SignCorrect}_{+}\quad \frac{\langle b, f\rangle \in S,\quad \alpha(b) \geq 0,\quad \alpha(f) \neq 1}{\alpha := \mathit{update}(\alpha, f, 1)}$$

$$\mathsf{SignSplit}\quad \frac{\langle b, f\rangle \in S}{\begin{array}{ll} u(b) := \min(u(b), -\epsilon), & l(b) := \max(l(b), 0), \\ l(f) := \max(l(f), -1), & l(f) := \max(l(f), 1), \\ u(f) := \min(u(f), -1) & u(f) := \min(u(f), 1) \end{array}}$$

$$\mathsf{Success}\quad \frac{\begin{array}{c}\forall x \in \mathcal{X}.\ l(x) \leq \alpha(x) \leq u(x), \\ \forall \langle b, f\rangle \in S.\ \alpha(f) = \mathrm{sign}(\alpha(b)),\quad \forall \langle b, f\rangle \in R.\ \alpha(f) = \mathrm{ReLU}(\alpha(b))\end{array}}{\mathtt{SAT}}$$

Fig. 4: The extended Reluplex derivation rules, with support for sign constraints.

straints and ReLU constraints are unchanged — the approach is modular and extensible in that sense, as each type of constraint is addressed separately. In Fig. 4, we depict new derivation rules, capable of addressing sign constraints. The $\mathsf{SignCorrect}_{-}$ and $\mathsf{SignCorrect}_{+}$ rules allow us to adjust the assignment of $f$ to account for the current assignment of $b$ — i.e., set $f$ to $-1$ if $b$ is negative, and to 1 otherwise. The SignSplit is used for performing a case split on a sign constraint, introducing a disjunction for enforcing that either $b$ is non-negative ($l(b) \geq 0$) and $f = 1$, or $b$ is negative ($u(b) \leq -\epsilon$; epsilon is a small positive constant, chosen to reflect the desired precision) and $f = -1$. Finally, the Success rule *replaces* the one from Fig. 3: it requires that all linear, ReLU and sign constraints be satisfied simultaneously.

We demonstrate this process with a simple example. Observe again the toy example for Fig. 2, the pre-condition $P = (1 \leq v_1^1 \leq 2) \wedge (-1 \leq v_1^2 \leq 1)$, and the post-condition $Q = (v_5^1 \leq 5)$. Our goal is to find an assignment to the variables $\{v_1^1, v_1^2, v_2^1, v_3^1, v_4^1, v_5^1\}$ that satisfies $P$, $Q$, and also the constraints imposed by the BNN itself, namely the weighted sums $v_2^1 = v_1^1 - v_1^2 + 1$, $v_3^1 = 0.5v_2^1$, and $v_5^1 = 2v_4^1$, and the sign constraint $v_4^1 = \mathrm{sign}(v_3^1)$.

| variable | $v_1^1$ | $v_1^2$ | $v_2^1$ | $v_3^1$ | $v_4^1$ | $v_5^1$ |
|---|---|---|---|---|---|---|
| assignment 1 | 1 | 0 | 2 | 1 | −1 | −2 |
| assignment 2 | 1 | 0 | 2 | 1 | **1** | −2 |
| assignment 3 | 1 | 0 | 2 | 1 | 1 | **2** |

Fig. 5: An iterative solution for a BNN verification query.

Initially, we invoke derivation rules that address the linear constraints (see [33]), and come up with an assignment that satisfies them, depicted as assignment 1 in Fig. 5. However, this assignment violates the sign constraint: $v_4^1 = -1 \neq \mathrm{sign}(v_3^1) = \mathrm{sign}(1) = 1$. We can thus invoke the $\mathsf{SignCorrect}_{+}$ rule, which adjusts the assignment, leading to assignment 2 in the figure. The sign constraint is now satisfied, but the linear constraint $v_5^1 = 2v_4^1$ is violated. We thus let the solver correct the linear constraints again, this time obtaining assignment 3 in the figure, which satisfies all constraints. The Success rule now applies, and we return `SAT` and the satisfying variable assignment.

The above-described calculus is sound and complete (assuming the $\epsilon$ used in the SignSplit rule is sufficiently small): when it answers `SAT` or `UNSAT`, that statement is correct, and for any input query there is a sequence of derivation steps that will lead to either SAT or UNSAT. The proof is quite similar to that of the original Reluplex procedure [33], and is omitted. A naive strategy that will always lead to termination is to apply the SignSplit rule to saturation; this effectively transforms the problem into an (exponentially long) sequence of linear programs. Then, each of these linear programs can be solved quickly (linear programming is known to be in P). However, this strategy is typically quite slow. In the next section we discuss how many of these case splits can be avoided by applying multiple optimizations.

## 4 Optimizations

**Weighted Sum Layer Elimination.** The SMT-based approach introduces a new variable for each node in a weighted sum layer, and an equation to express that node's value as a weighted sum of nodes from the preceding layer. In BNNs, we often encounter consecutive weighted sum layers — specifically because of the binary block structure, in which a weighted sum layer is followed by a batch normalization layer, which is also encoded as weighted sum layer. Thus, a straightforward way to reduce the number of variables and equations, and hence to expedite the solution process, is to combine two consecutive weighted sum layers into a single layer. Specifically, the original layers can be regarded as transforming input $x$ into $y = W_2(W_1 \cdot x + B_1) + B_2$, and the simplification as computing $y = W_3 \cdot x + B_3$, where $W_3 = W_2 \cdot W_1$ and $B_3 = W_2 \cdot B_1 + B_2$. An illustration appears in Fig. 6 (for simplicity, all bias values are assumed to be 0).

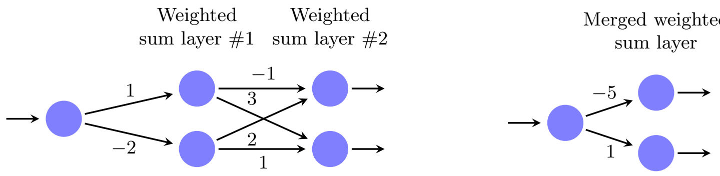

Fig. 6: On the left, a (partial) DNN with two consecutive weighted sum layers. On the right, an equivalent DNN with these two layers merged into one.

**LP Relaxation.** Given a constraint $f = \text{sign}(b)$, it is beneficial to deduce tighter bounds on the $b$ and $f$ variables — especially if these tighter bounds fix the constraints into one of its linear phases. We thus introduce a preprocessing phase, prior to the invocation of our enhanced Reluplex procedure, in which tighter bounds are computed by invoking a linear programming (LP) solver.

The idea, inspired by similar relaxations for ReLU nodes [14, 49], is to over-approximate each constraint in the network, including sign constraints, as a set of linear constraints. Then, for every variable $v$ in the encoding, an LP solver is used to compute an upper bound $u$ (by maximizing) and a lower bound $l$ (by minimizing) for $v$. Because the LP encoding is an over-approximation, $v$ is indeed within the range $[l, u]$ for any input to the network.

Let $f = \text{sign}(b)$, and suppose we initially know that $l \leq b \leq u$. The linear over-approximation that we introduce for $f$ is a trapezoid (see Fig. 7), with the following edges: (i) $f \leq 1$; (ii) $f \geq -1$; (iii) $f \leq \frac{2}{-l} \cdot b + 1$; and (iv) $f \geq \frac{2}{u} \cdot b - 1$. It is straightforward to show that these four equations form the smallest convex polytope containing the values of $f$.

We demonstrate this process on the simple BNN depicted on the left-hand side of Fig. 7. Suppose we know that the input variable, $x$, is bounded in the range $-1 \leq x \leq 1$, and we wish to compute a lower bound for $y$. Simple, interval-arithmetic based bound propagation [33] shows that $b_1 = 3x+1$ is bounded in the range $-2 \leq b_1 \leq 4$, and similarly that $b_2 = -4x+2$ is in the range $-2 \leq b_2 \leq 6$. Because neither $b_1$ nor $b_2$ are strictly negative or positive, we only know that $-1 \leq f_1, f_2 \leq 1$, and so the best bound obtainable for $y$ is $y \geq -2$. However, by formulating the LP relaxation of the problem (right-hand side of Fig. 7), we get the optimal solution $x = -\frac{1}{3}, b_1 = 0, b_2 = \frac{10}{3}, f_1 = -1, f_2 = \frac{1}{9}, y = -\frac{8}{9}$, implying the tighter bound $y \geq -\frac{8}{9}$.

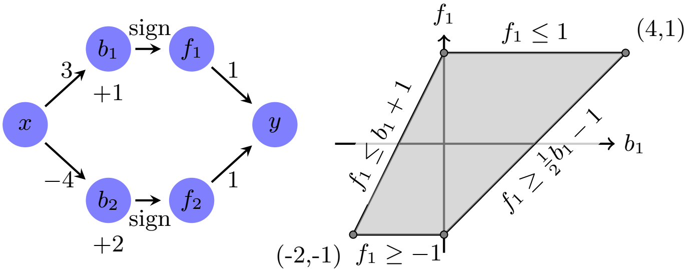

**minimize** $y$ **s.t.**:
$-1 \leq x \leq 1$
$b_1 = 3x + 1$
$b_2 = -4x + 2$
$y = f_1 + f_2$

$-1 \leq f_1 \leq 1$
$-1 \leq f_2 \leq 1$
$\frac{b_1}{2} - 1 \leq f_1 \leq b_1 + 1$
$\frac{b_2}{3} - 1 \leq f_2 \leq b_2 + 1$

Fig. 7: A simple BNN (left), the trapezoid relaxation of $f_1 = \text{sign}(b_1)$ (center), and its LP encoding (right). The trapezoid relaxation of $f_2$ is not depicted.

The aforementioned linear relaxation technique is effective but expensive — because it entails invoking the LP solver twice for each neuron in the BNN encoding. Consequently, in our tool, the technique is applied only once per query, as a preprocessing step. Later, during the search procedure, we apply a related but more lightweight technique, called *symbolic bound tightening* [52], which we enhanced to support sign constraints.

**Symbolic Bound Tightening.** In symbolic bound tightening, we compute for each neuron $v$ a symbolic lower bound $sl(x)$ and a symbolic upper bound $su(x)$, which are linear combinations of the input neurons. Upper and lower bounds can then be derived from their symbolic counterparts using simple interval arithmetic. For example, suppose the network's input nodes are $x_1$ and

$x_2$, and that for some neuron $v$ we have:

$$sl(v) = 5x_1 - 2x_2 + 3, \quad su(v) = 3x_1 + 4x_2 - 1$$

and that the currently known bounds are $x_1 \in [-1, 2], x_2 \in [-1, 1]$ and $v \in [-2, 11]$. Using the symbolic bounds and the input bounds, we can derive that the upper bound of $v$ is at most $6 + 4 - 1 = 9$, and that its lower bound is at least $-5 - 2 + 3 = -4$. In this case, the upper bound we have discovered for $v$ is tighter than the previous one, and so we can update $v$'s range to be $[-2, 9]$.

The symbolic bound expressions are propagated layer by layer [52]. Propagation through weighted sum layers is straightforward: the symbolic bounds are simply multiplied by the respective edge weights and summed up. Efficient approaches for propagations through ReLU layers have also been proposed [51]. Our contribution here is an extension of these techniques for propagating symbolic bounds also through sign layers. The approach again uses a trapezoid, although a more coarse one — so that we can approximate each neuron from above and below using a single linear expression. More specifically, for $f = \text{sign}(b)$ with $b \in [l, u]$ and previously-computed symbolic bounds $su(b)$ and $sl(b)$, the symbolic bounds for $f$ are given by:

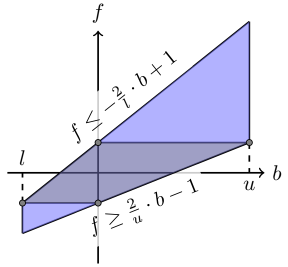

Fig. 8: Symbolic bounds for f=sign(b).

$$sl(f) = \frac{2}{u} \cdot sl(b) - 1, \quad su(f) = -\frac{2}{l} \cdot su(b) + 1$$

An illustration appears in Fig. 8. The blue trapezoid is the relaxation we use for the symbolic bound computation, whereas the gray trapezoid is the one used for the LP relaxation discussed previously. The blue trapezoid is larger, and hence leads to looser bounds than the gray trapezoid; but it is computationally cheaper to compute and use, and our evaluation demonstrates its usefulness.

**Polarity-based Splitting.** The Marabou framework supports a parallelized solving mode, using the Split-and-Conquer (S&C) algorithm [54]. At a high level, S&C partitions a verification query $\phi$ into a set of sub-queries $\Phi := \{\phi_1, ...\phi_n\}$, such that $\phi$ and $\bigvee_{\phi' \in \Phi} \phi'$ are equi-satisfiable, and handles each sub-query independently. Each sub-query is solved with a timeout value; and if that value is reached, the sub-query is again split into additional sub-queries, and each is solved with a greater timeout value. The process repeats until one of the sub-queries is determined to be `SAT`, or until all sub-queries are proven `UNSAT`.

One Marabou strategy for creating sub-queries is by splitting the ranges of input neurons. For example, if in query $\phi$ an input neuron $x$ is bounded in the range $x \in [0, 4]$ and $\phi$ times out, it might be split into $\phi_1$ and $\phi_2$ such that $x \in [0, 2]$ in $\phi_1$ and $x \in [2, 4]$ in $\phi_2$. This strategy is effective when the neural network being verified has only a few input neurons.

Another way to create sub-queries is to perform case-splits on piecewise-linear constraints — sign constraints, in our case. For instance, given a verification query $\phi := \phi' \wedge f = \text{sign}(b)$, we can partition it into $\phi^- := \phi' \wedge b < 0 \wedge f = -1$ and $\phi^+ := \phi' \wedge b \geq 0 \wedge f = 1$. Note that $\phi$ and $\phi^+ \vee \phi^-$ are equi-satisfiable.

The heuristics for picking which sign constraint to split on have a significant impact on the difficulty of the resulting sub-problems [54]. Specifically, it is desirable that the sub-queries be *easier* than the original query, and also that they be *balanced* in terms of runtime — i.e., we wish to avoid the case where $\phi_1$ is very easy and $\phi_2$ is very hard, as that makes poor use of parallel computing resources. To create easier sub-problems, we propose to split on sign constraints that occur in the earlier layers of the BNN, as that leads to efficient bound propagation when combined with our symbolic bound tightening mechanism. To create balanced sub-problems, we use a metric called *polarity*, which was proposed in [54] for ReLUs and is extended here to support sign constraints.

**Definition 1.** *Given a sign constraint $f = sign(b)$, and the bounds $l \leq b \leq u$, where $l < 0$, and $u > 0$, the polarity of the sign constraint is defined as $p = \frac{u+l}{u-l}$.*

Intuitively, the closer the polarity is to 0, the more balanced the resulting queries will be if we perform a case-split on this constraint. For example, if $\phi = \phi' \wedge -10 \leq b \leq 10$ and we create $\phi_1 = \phi' \wedge -10 \leq b < 0$, $\phi_2 = \phi' \wedge 0 \leq b \leq 10$, then queries $\phi_1$ and $\phi_2$ are roughly balanced. However, if initially $-10 \leq b \leq 1$, we obtain $\phi_1 = \phi' \wedge -10 \leq b < 0$ and $\phi_2 = \phi' \wedge 0 \leq b \leq 1$. In this case, $\phi_2$ might prove significantly easier than $\phi_1$ because the smaller range of $b$ in $\phi_2$ could lead to very effective bound tightening. Consequently, we use a heuristic that picks the sign constraint with the smallest polarity among the first $k$ candidates (in topological order), where $k$ is a configurable parameter. In our experiments, we empirically selected $k = 5$.

### 5 Implementation

We implemented our approach as an extension to Marabou [34], which is an open-source, freely available SMT-based DNN verification framework [2]. Marabou implements the Reluplex algorithm, but with multiple extensions and optimizations — e.g., support for additional activation functions, deduction methods, and parallelization [54]. It has been used for a variety of verification tasks, such as network simplification [19] and optimization [47], verification of video streaming protocols [35], DNN modification [20], adversarial robustness evaluation [9,22,32] verification of recurrent networks [28], and others. However, to date Marabou could not support sign constraints, and thus, could not be used to verify BNNs. Below we describe our main contributions to the code base. Our complete code is available as an artifact accompanying this paper [1], and has also been merged into the main Marabou repository [2].

**Basic Support for Sign Constraints (*SignConstraint.cpp*).** During execution, Marabou maintains a set of piecewise-linear constraints that are part of the query being solved. To support various activation functions, these constraints are represented using classes that inherit from the abstract *PiecewiseLinearConstraint* class. Here, we added a new sub-class, *SignConstraint*, that inherits from *PiecewiseLinearConstraint.* The methods of this class check whether the piecewise-linear sign constraint is satisfied, and in case it is not — which possible changes to the current assignment could fix the violation. This class' methods also extend Marabou's deduction mechanism for bound tightening.

**Input Interfaces for Sign Constraints (*MarabouNetworkTF.py*).** Marabou supports various input interfaces, most notable of which is the TensorFlow interface, which automatically translates a DNN stored in TensorFlow *protobuf* or *savedModel* formats into a Marabou query. As part of our extensions, we enhanced this interface so that it can properly handle BNNs and sign constraints. Additionally, users can create queries using Marabou's native C++ interface, by instantiating the *SignConstraint* class discussed previously.

**Network-Level Reasoner (*NetworkLevelReasoner.cpp, Layer.cpp, LPFormulator.cpp*).** The *Network-Level Reasoner* (*NLR*) is the part of Marabou that is aware of the topology of the neural network being verified, as opposed to just the individual constraints that comprise it. We extended Marabou's NLR to support sign constraints and implement the optimizations discussed in Section 4. Specifically, one extension that we added allows this class to identify consecutive weighted sum layers and merge them. Another extension creates a linear over-approximation of the network, including the trapezoid-shaped over-approximation of each sign constraint. As part of the symbolic bound propagation process, the NLR traverses the network, layer by layer, each time computing the symbolic bound expressions for each neuron in the current layer.

**Polarity-Based Splitting (*DnCManager.cpp*).** We extended the methods of this class, which is part of Marabou's S&C mechanism, to compute the polarity value of each sign constraint (see Definition 1), based on the current bounds.

### 6 Evaluation

All the benchmarks described in this section are included in our artifact, and are publicly available online [1].

**Strictly Binarized Networks.** We began by training a strictly binarized network over the MNIST digit recognition dataset.[4] This dataset includes 70,000 images of handwritten digits, each given as a $28 \times 28$ pixeled image, with normalized brightness values ranging from 0 to 1. The network that we trained has an input layer of size 784, followed by six binary blocks (four blocks of size 50,

[4] `http://yann.lecun.com/exdb/mnist/`

two blocks of size 10), and a final output layer with 10 neurons. Note that in the first block we omitted the sign layer in order to improve the network's accuracy.[5] The model was trained for 300 epochs using the *Larq* library [17] and the *Adam* optimizer [36], achieving 90% accuracy.

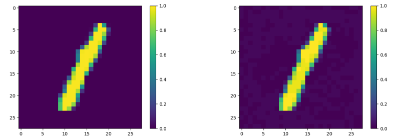

Fig. 9: An adversarial example for the MNIST network.

After training, we used Larq's export mechanism to save the trained network in a TensorFlow format, and then used our newly added Marabou interface to load it. For our verification queries, we first chose 500 samples from the test set which were classified correctly by the network. Then, we used these samples to formulate *adversarial robustness* queries [33,48]: queries that ask Marabou to find a slightly perturbed input which is misclassified by the network, i.e. is assigned a different label than the original. We formulated 500 queries, constructed from 50 queries for each of ten possible perturbation values $\delta \in \{0.1, 0.15, 0.2, 0.3, 0.5, 1, 3, 5, 10, 15\}$ in $L_\infty$ norm, one query per input sample. An `UNSAT` answer from Marabou indicates that no adversarial perturbation exists (for the specified $\delta$), whereas a `SAT` answer includes, as the counterexample, an actual perturbation that leads to misclassification. Such adversarial robustness queries are the most widespread verification benchmarks in the literature (e.g., [16,25,33,52]). An example appears in Fig. 9: the image on the left is the original, correctly classified as 1, and the image on the right is the perturbed image discovered by Marabou, misclassified as 3.

Through our experiments we set out to evaluate our tool's performance, and also measured the contribution of each of the features that we introduced: (i) weighted sum (ws) layer elimination; (ii) LP relaxation; (iii) symbolic bound tightening (sbt); and (iv) polarity-based splitting. We thus defined five configurations of the tool: the *all* category, in which all four features are enabled, and four *all-X* configurations for $X \in$ {*ws, lp, sbt, polarity*}, indicating that feature X is turned off and the other features are enabled. All five configurations utilized Marabou's parallelization features, except for *all-polarity* — where instead of polarity-based splitting we used Marabou's default splitting strategy, which splits the input domain in half in each step.

Fig. 10 depicts Marabou's results using each of the five configurations. Each experiment was run on an Intel Xeon E5-2637 v4 CPUs machine, running Ubuntu 16.04 and using eight cores, with a wall-clock timeout of 5,000 seconds. Most notably, the results show the usefulness of polarity-based splitting when compared to Marabou's default splitting strategy: whereas the *all-polarity* configuration only solved 218 instances, the *all* configuration solved 458. It also shows that the weighted sum layer elimination feature significantly improves performance, from 436 solved instances in *all-ws* to 458 solved instances in *all*, and with significantly faster solving speed. With the remaining two features, namely LP

[5] This is standard practice; see `https://docs.larq.dev/larq/guides/bnn-architecture/`

relaxations and symbolic bound tightening, the results are less clear: although the *all-lp* and *all-sbt* configurations both slightly outperform the *all* configuration, indicating that these two features slowed down the solver, we observe that for many instances they do lead to an improvement; see Fig. 11. Specifically, on UNSAT instances, the *all* configuration was able to solve one more benchmark than either *all-lp* or *all-sbt*; and it strictly outperformed *all-lp* on 13% of the instances, and *all-sbt* on 21% of the instances. Gaining better insights into the causes for these differences is a work in progress.

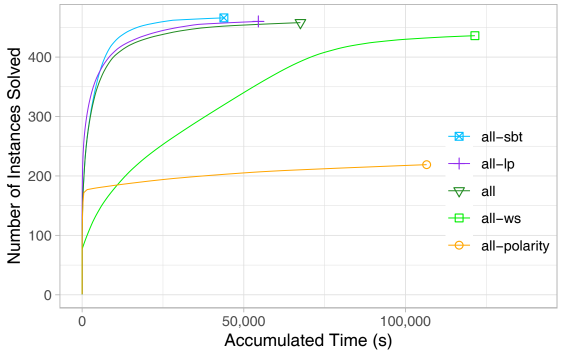

Fig. 10: Running the five configurations of Marabou on the MNIST BNN.

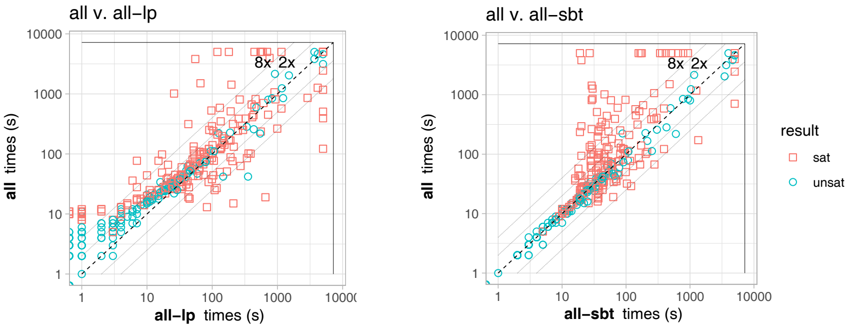

Fig. 11: Evaluating the LP relaxation and symbolic bound tightening features.

**XNOR-Net.** XNOR-Net [45] is a BNN architecture for image recognition networks. XNOR-Nets consist of a series of *binary convolution* blocks, each containing a sign layer, a convolution layer, and a max-pooling layer (here, we regard convolution layers as a specific case of weighted sum layers). We constructed such a network with two binary convolution blocks: the first block has three layers, including a convolution layer with three filters, and the second block has four layers, including a convolution layer with two filters. The two binary convolution blocks are followed by a batch normalization layer and a fully-connected weighted sum layer (10 neurons) for the network's output, as depicted in Fig. 12. Our network was trained on the Fashion-MNIST dataset, which includes 70,000 images from ten different clothing categories [55], each given as a $28 \times 28$ pixeled image. The model was trained for 30 epochs, and achieved a modest accuracy of 70.97%.

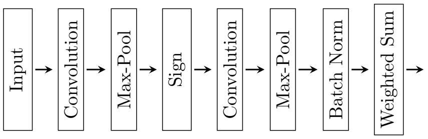

Fig. 12: The XNOR-Net architecture of our network.

For our verification queries, we chose 300 correctly classified samples from the test set, and used them to formulate adversarial robustness queries. Each query was formulated using one sample and a perturbation value $\delta \in \{0.05, 0.1, 0.15, 0.2, 0.25, 0.3\}$ in $L_\infty$ norm. Fig. 13 depicts the adversarial image that Marabou produced for one of these queries. The image on the left is a correctly classified image of a shirt, and the image on the right is the perturbed image, now misclassified as a coat.

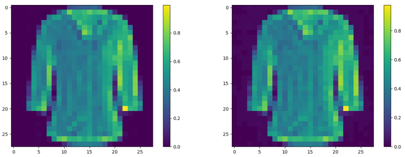

Fig. 13: An original image (left) and its perturbed, misclassified image (right).

Based on the results from the previous set of experiments, we used Marabou with weighted sum layer elimination and polarity-based splitting turned on, but with symbolic bound tightening and LP relaxation turned off. Each experiment ran on an Intel Xeon E5-2637 v4 machine, using eight cores and a wall-clock timeout of 7,200 seconds. The results are depicted in Table 1. The results demonstrate that `UNSAT` queries tended to be solved significantly faster than `SAT` ones, indicating that Marabou's search procedure for these cases needs further optimization. Overall, Marabou was able to solve 203 out of 300 queries. To the best of our knowledge, this is the first effort to formally verify an XNOR-Net. We note that these results demonstrate the usefulness of an SMT-based approach for BNN verification, as it allows the verification of DNNs with multiple types of activation functions, such as a combination of sign and max-pooling.

### 7 Related Work

DNNs have become pervasive in recent years, and the discovery of various faults and errors has given rise to multiple approaches for verifying them. These in-

Table 1: Marabou's performance on the XNOR-Net queries.

| $\delta$ | SAT | | UNSAT | | |
|---|---|---|---|---|---|
| | # Solved | Avg. Time (s) | # Solved | Avg. Time (s) | # Timeouts |
| 0.05 | 15 | 909.13 | 23 | 4.96 | 12 |
| 0.1 | 15 | 1,627.67 | 20 | 12.15 | 15 |
| 0.15 | 9 | 1,113.33 | 29 | 5 | 12 |
| 0.2 | 10 | 1,387.7 | 24 | 4.96 | 16 |
| 0.25 | 9 | 1,426 | 22 | 4.91 | 19 |
| 0.3 | 7 | 1,550.86 | 20 | 26.75 | 23 |
| Total | 65 | 1,317.52 | 138 | 9.16 | 97 |

clude various SMT-based approaches (e.g., [25, 33, 34, 38]), approaches based on LP and MILP solvers (e.g., [8, 14, 41, 49]), approaches based on symbolic interval propagation or abstract interpretation (e.g., [16,50,52,53]), abstraction-refinement (e.g., [3, 15]), and many others. Most of these lines of work have focused on non-quantized DNNs. Verification of quantized DNNs is PSPACE-hard [24], and requires different tools than the ones used for their non-quantized counterparts [18]. Our technique extends an existing line of SMT-based verifiers to support also the sign activation functions needed for verifying BNNs; and these new activations can be combined with various other layers.

Work to date on the verification of BNNs has relied exclusively on reducing the problem to Boolean satisfiability, and has thus been limited to the strictly binarized case [11,29,43,44]. Our approach, in contrast, can be applied to binarized neural networks that include activation functions beyond the sign function, as we have demonstrated by verifying an XNOR-Net. Comparing the performance of Marabou and the SAT-based approaches is left for future work.

### 8 Conclusion

BNNs are a promising avenue for leveraging deep learning in devices with limited resources. However, it is highly desirable to verify their correctness prior to deployment. Here, we propose an SMT-based verification approach that enables the verification of BNNs. This approach, which we have implemented as part of the Marabou framework [2], seamlessly integrates with the other components of the SMT solver in a modular way. Using Marabou, we have verified, for the first time, a network that uses both binarized and non-binarized layers. In the future, we plan to improve the scalability of our approach, by enhancing it with stronger bound deduction capabilities, based on abstract interpretation [16].

**Acknowledgements.** We thank Nina Narodytska, Kyle Julian, Kai Jia, Leon Overweel and the Plumerai research team for their contributions to this project. The project was partially supported by the Israel Science Foundation (grant number 683/18), the Binational Science Foundation (grant number 2017662), the National Science Foundation (grant number 1814369), and the Center for Interdisciplinary Data Science Research at The Hebrew University of Jerusalem.

## References

[1] Artifact repository. https://github.com/guyam2/BNN_Verification_Artifact.

[2] Marabou repository. https://github.com/NeuralNetworkVerification/Marabou.

[3] P. Ashok, V. Hashemi, J. Kretinsky, and S. Mühlberger. DeepAbstract: Neural Network Abstraction for Accelerating Verification. In *Proc. 18th Int. Symposium on Automated Technology for Verification and Analysis (ATVA)*, 2020.

[4] P. Bacchus, R. Stewart, and E. Komendantskaya. Accuracy, Training Time and Hardware Efficiency Trade-Offs for Quantized Neural Networks on FPGAs. In *Proc. 16th Int. Symposium on Applied Reconfigurable Computing (ARC)*, pages 121–135, 2020.

[5] C. Barrett and C. Tinelli. *Satisfiability modulo theories*. Springer, 2018.

[6] O. Bastani, Y. Ioannou, L. Lampropoulos, D. Vytiniotis, A. Nori, and A. Criminisi. Measuring Neural Net Robustness with Constraints. In *Proc. 30th Conf. on Neural Information Processing Systems (NIPS)*, 2016.

[7] M. Bojarski, D. Del Testa, D. Dworakowski, B. Firner, B. Flepp, P. Goyal, L. Jackel, M. Monfort, U. Muller, J. Zhang, X. Zhang, J. Zhao, and K. Zieba. End to End Learning for Self-Driving Cars, 2016. Technical Report. http://arxiv.org/abs/1604.07316.

[8] R. Bunel, I. Turkaslan, P. Torr, P. Kohli, and P. Mudigonda. A Unified View of Piecewise Linear Neural Network Verification. In *Proc. 32nd Conf. on Neural Information Processing Systems (NeurIPS)*, pages 4795–4804, 2018.

[9] N. Carlini, G. Katz, C. Barrett, and D. Dill. Provably Minimally-Distorted Adversarial Examples, 2017. Technical Report. https://arxiv.org/abs/1709.10207.

[10] H. Chen, L. Zhuo, B. Zhang, X. Zheng, J. Liu, R. Ji, D. D., and G. Guo. Binarized Neural Architecture Search for Efficient Object Recognition, 2020. Technical Report. http://arxiv.org/abs/2009.04247.

[11] C.-H. Cheng, G. Nührenberg, C.-H. Huang, and H. Ruess. Verification of Binarized Neural Networks via Inter-Neuron Factoring, 2017. Technical Report. http://arxiv.org/abs/1710.03107.

[12] D. Ciregan, U. Meier, and J. Schmidhuber. Multi-Column Deep Neural Networks for Image Classification. In *Proc. IEEE Conf. on Computer Vision and Pattern Recognition (CVPR)*, pages 3642–3649, 2012.

[13] G. Dantzig. *Linear Programming and Extensions*. Princeton University Press, 1963.

[14] R. Ehlers. Formal Verification of Piece-Wise Linear Feed-Forward Neural Networks. In *Proc. 15th Int. Symp. on Automated Technology for Verification and Analysis (ATVA)*, pages 269–286, 2017.

[15] Y. Elboher, J. Gottschlich, and G. Katz. An Abstraction-Based Framework for Neural Network Verification. In *Proc. 32nd Int. Conf. on Computer Aided Verification (CAV)*, pages 43–65, 2020.

[16] T. Gehr, M. Mirman, D. Drachsler-Cohen, E. Tsankov, S. Chaudhuri, and M. Vechev. AI2: Safety and Robustness Certification of Neural Networks with Abstract Interpretation. In *Proc. 39th IEEE Symposium on Security and Privacy (S&P)*, 2018.

[17] L. Geiger and P. Team. Larq: An Open-Source Library for Training Binarized Neural Networks. *Journal of Open Source Software*, 5(45):1746, 2020.

[18] M. Giacobbe, T. Henzinger, and M. Lechner. How Many Bits Does it Take to Quantize Your Neural Network? In *Proc. 26th Int. Conf. on Tools and Algorithms for the Construction and Analysis of Systems (TACAS)*, pages 79–97, 2020.

[19] S. Gokulanathan, A. Feldsher, A. Malca, C. Barrett, and G. Katz. Simplifying Neural Networks using Formal Verification. In *Proc. 12th NASA Formal Methods Symposium (NFM)*, pages 85–93, 2020.

[20] B. Goldberger, Y. Adi, J. Keshet, and G. Katz. Minimal Modifications of Deep Neural Networks using Verification. In *Proc. 23rd Int. Conf. on Logic for Programming, Artificial Intelligence and Reasoning (LPAR)*, pages 260–278, 2020.

[21] I. Goodfellow, Y. Bengio, A. Courville, and Y. Bengio. *Deep learning*, volume 1. MIT press Cambridge, 2016.

[22] D. Gopinath, G. Katz, C. Pǎsǎreanu, and C. Barrett. DeepSafe: A Data-driven Approach for Assessing Robustness of Neural Networks. In *Proc. 16th. Int. Symposium on on Automated Technology for Verification and Analysis (ATVA)*, pages 3–19, 2018.

[23] S. Han, H. Mao, and W. Dally. Deep Compression: Compressing Deep Neural Networks with Pruning, Trained Quantization and Huffman Coding. In *Proc. 4th Int. Conf. on Learning Representations (ICLR)*, 2016.

[24] T. Henzinger, M. Lechner, and D. Zikelic. Scalable Verification of Quantized Neural Networks (Technical Report), 2020. Technical Report. https://arxiv.org/abs/2012.08185.

[25] X. Huang, M. Kwiatkowska, S. Wang, and M. Wu. Safety Verification of Deep Neural Networks. In *Proc. 29th Int. Conf. on Computer Aided Verification (CAV)*, pages 3–29, 2017.

[26] I. Hubara, M. Courbariaux, D. Soudry, R. El-Yaniv, and Y. Bengio. Binarized Neural Networks. In *Proc. 30th Conf. on Neural Information Processing Systems (NIPS)*, pages 4107–4115, 2016.

[27] I. Hubara, M. Courbariaux, D. Soudry, R. El-Yaniv, and Y. Bengio. Quantized Neural Networks: Training Neural Networks with Low Precision Weights and Activations. *The Journal of Machine Learning Research*, 18(1):6869–6898, 2017.

[28] Y. Jacoby, C. Barrett, and G. Katz. Verifying Recurrent Neural Networks using Invariant Inference. In *Proc. 18th Int. Symposium on Automated Technology for Verification and Analysis (ATVA)*, 2020.

[29] K. Jia and M. Rinard. Efficient Exact Verification of Binarized Neural Networks, 2020. Technical Report. http://arxiv.org/abs/2005.03597.

[30] K. Julian, J. Lopez, J. Brush, M. Owen, and M. Kochenderfer. Policy Compression for Aircraft Collision Avoidance Systems. In *Proc. 35th Digital Avionics Systems Conf. (DASC)*, pages 1–10, 2016.

[31] G. Katz, C. Barrett, D. Dill, K. Julian, and M. Kochenderfer. Reluplex: An Efficient SMT Solver for Verifying Deep Neural Networks. In *Proc. 29th Int. Conf. on Computer Aided Verification (CAV)*, pages 97–117, 2017.

[32] G. Katz, C. Barrett, D. Dill, K. Julian, and M. Kochenderfer. Towards Proving the Adversarial Robustness of Deep Neural Networks. In *Proc. 1st Workshop on Formal Verification of Autonomous Vehicles (FVAV)*, pages 19–26, 2017.

[33] G. Katz, C. Barrett, D. Dill, K. Julian, and M. Kochenderfer. Reluplex: a Calculus for Reasoning about Deep Neural Networks, 2021. Submitted, preprint avaialble upon request.

[34] G. Katz, D. Huang, D. Ibeling, K. Julian, C. Lazarus, R. Lim, P. Shah, S. Thakoor, H. Wu, A. Zeljić, D. Dill, M. Kochenderfer, and C. Barrett. The Marabou Framework for Verification and Analysis of Deep Neural Networks. In *Proc. 31st Int. Conf. on Computer Aided Verification (CAV)*, pages 443–452, 2019.

[35] Y. Kazak, C. Barrett, G. Katz, and M. Schapira. Verifying Deep-RL-Driven Systems. In *Proc. 1st ACM SIGCOMM Workshop on Network Meets AI & ML (NetAI)*, pages 83–89, 2019.

[36] D. Kingma and J. Ba. Adam: a Method for Stochastic Optimization, 2014. Technical Report. http://arxiv.org/abs/1412.6980.

[37] A. Krizhevsky, I. Sutskever, and G. Hinton. Imagenet Classification with Deep Convolutional Neural Networks. In *Proc. 26th Conf. on Neural Information Processing Systems (NIPS)*, pages 1097–1105, 2012.

[38] L. Kuper, G. Katz, J. Gottschlich, K. Julian, C. Barrett, and M. Kochenderfer. Toward Scalable Verification for Safety-Critical Deep Networks, 2018. Technical Report. https://arxiv.org/abs/1801.05950.

[39] S. Lai, L. Xu, K. Liu, and J. Zhao. Recurrent Convolutional Neural Networks for Text Classification. In *Proc. 29th AAAI Conf. on Artificial Intelligence*, 2015.

[40] D. Lin, S. Talathi, and S. Annapureddy. Fixed Point Quantization of Deep Convolutional Networks. In *Proc. 33rd Int. Conf. on Machine Learning (ICML)*, pages 2849–2858, 2016.

[41] A. Lomuscio and L. Maganti. An Approach to Reachability Analysis for Feed-Forward ReLU Neural Networks, 2017. Technical Report. http://arxiv.org/abs/1706.07351.

[42] P. Molchanov, S. Tyree, T. Karras, T. Aila, and J. Kautz. Pruning Convolutional Neural Networks for Resource Efficient Inference, 2016. Technical Report. http://arxiv.org/abs/1611.06440.

[43] N. Narodytska, S. Kasiviswanathan, L. Ryzhyk, M. Sagiv, and T. Walsh. Verifying Properties of Binarized Deep Neural Networks, 2017. Technical Report. http://arxiv.org/abs/1709.06662.

[44] N. Narodytska, H. Zhang, A. Gupta, and T. Walsh. In Search for a SAT-friendly Binarized Neural Network Architecture. In *Proc. 7th Int. Conf. on Learning Representations (ICLR)*, 2019.

[45] M. Rastegari, V. Ordonez, J. Redmon, and A. Farhadi. XNOR-Net: Imagenet Classification using Binary Convolutional Neural Networks. In *Proc. 14th European Conf. on Computer Vision (ECCV)*, pages 525–542, 2016.

[46] K. Simonyan and A. Zisserman. Very Deep Convolutional Networks for Large-Scale Image Recognition. In *Proc. 3rd Int. Conf. on Learning Representations (ICLR)*, 2015.

[47] C. Strong, H. Wu, A. Zeljić, K. Julian, G. Katz, C. Barrett, and M. Kochenderfer. Global Optimization of Objective Functions Represented by ReLU networks, 2020. Technical Report. http://arxiv.org/abs/2010.03258.

[48] C. Szegedy, W. Zaremba, I. Sutskever, J. Bruna, D. Erhan, I. Goodfellow, and R. Fergus. Intriguing Properties of Neural Networks, 2013. Technical Report. http://arxiv.org/abs/1312.6199.

[49] V. Tjeng, K. Xiao, and R. Tedrake. Evaluating Robustness of Neural Networks with Mixed Integer Programming. In *Proc. 7th Int. Conf. on Learning Representations (ICLR)*, 2019.

[50] H. Tran, S. Bak, and T. Johnson. Verification of Deep Convolutional Neural Networks Using ImageStars. In *Proc. 32nd Int. Conf. on Computer Aided Verification (CAV)*, pages 18–42, 2020.

[51] S. Wang, K. Pei, J. Whitehouse, J. Yang, and S. Jana. Efficient Formal Safety Analysis of Neural Networks, 2018. Technical Report. https://arxiv.org/abs/1809.08098.

[52] S. Wang, K. Pei, J. Whitehouse, J. Yang, and S. Jana. Formal Security Analysis of Neural Networks using Symbolic Intervals. In *Proc. 27th USENIX Security Symposium*, pages 1599–1614, 2018.

[53] T.-W. Weng, H. Zhang, H. Chen, Z. Song, C.-J. Hsieh, D. Boning, I. Dhillon, and L. Daniel. Towards Fast Computation of Certified Robustness for ReLU Networks, 2018. Technical Report. http://arxiv.org/abs/1804.09699.

[54] H. Wu, A. Ozdemir, A. Zeljić, A. Irfan, K. Julian, D. Gopinath, S. Fouladi, G. Katz, C. Păsăreanu, and C. Barrett. Parallelization Techniques for Verifying Neural Networks. In *Proc. 20th Int. Conf. on Formal Methods in Computer-Aided Design (FMCAD)*, pages 128–137, 2020.

[55] H. Xiao, K. Rasul, and R. Vollgraf. Fashion-Mnist: a Novel Image Dataset for Benchmarking Machine Learning Algorithms, 2017. Technical Report. http://arxiv.org/abs/1708.07747.

[56] J. Yang, X. Shen, J. Xing, X. Tian, H. Li, B. Deng, J. Huang, and X.-S. Hua. Quantization Networks. In *Proc. IEEE Conf. on Computer Vision and Pattern Recognition (CVPR)*, pages 7308–7316, 2019.

[57] Y. Zhou, S.-M. Moosavi-Dezfooli, N.-M. Cheung, and P. Frossard. Adaptive Quantization for Deep Neural Network, 2017. Technical Report. http://arxiv.org/abs/1712.01048.

